# Decellularized lymph node sections with preserved extracellular matrix for stromal cell culture

**DOI:** 10.1038/s41598-025-28782-0

**Published:** 2025-11-24

**Authors:** Estefania Esparza, Leonor N. Teles, Alisa Fedotova, Noa Dehaseth, Mira Sayegh, Ana V. Hernandez, Lucy Y. Ho, Noel M. Ziebarth, Alice A. Tomei

**Affiliations:** 1 Department of Biomedical Engineering, University of Miami, Coral Gables, FL 33146 USA; 2 Diabetes Research Institute, University of Miami Miller School of Medicine, Miami, FL 33136 USA; 3 Department of Microbiology and Immunology, University of Miami Miller School of Medicine, Miami, FL 33136 USA

**Keywords:** Decellularization, Extracellular matrix, Lymphoid organs, 3D cell culture, Fibroblastic reticular cells, Biological techniques, Biotechnology, Cell biology

## Abstract

The lymph node (LN) extracellular matrix (ECM) is produced by stromal cells like fibroblastic reticular cells (FRCs) and supports adaptive immunity by guiding immune cell interactions. Disruption of this ECM in cancer and chronic inflammation has been shown to promote disease progression. While interactions between cells and the LN ECM are critical for immunity, they remain difficult to study due to limitations in current models and reliance on animal studies. To address this, LNs could be decellularized to generate cell-free scaffolds that are subsequently reseeded with cells to study how the native LN microenvironment influences cellular behavior. Existing whole-organ decellularization methods preserve ECM features but yield dense scaffolds that restrict uniform cell seeding, limit nutrient diffusion, and hinder imaging analyses. Here, we present a protocol that combines vibratome sectioning (200-μm slices) with detergent decellularization (0.1% SDS and 1% Triton-X) to generate thin LN slices from mouse and human tissues. Decellularized LNs had comparable collagen and GAG concentrations to native tissue, and immunofluorescence staining showed the presence of other ECM proteins. Decellularized sections sustained 21-day FRC culture, enabled FRC-T cell co-culture, and supported high-resolution imaging and flow cytometric analyses, revealing altered gp38 and PDGFRα expression in FRCs relative to 2D culture.

**Supplementary Information:**

The online version contains supplementary material available at 10.1038/s41598-025-28782-0.

## Introduction

Lymph nodes (LNs) are essential organs of the immune system where adaptive immune responses are initiated and regulated^1^. Within the LN paracortex, fibroblastic reticular cells (FRCs), a specialized stromal cell population, produce a network of extracellular matrix (ECM) fibers that guide immune cell migration, activation and interactions^2–5^. This highly organized ECM microenvironment is composed of collagens, glycosaminoglycans (GAGs), proteoglycans, and glycoproteins^6,7^. Collagen provides structural support and tensile strength^8^, while glycoproteins like laminin and fibronectin form basement membranes that facilitate cell adhesion and signaling through integrin-binding domains^5,9^. GAGs such as hyaluronic acid (HA) support cell trafficking and motility^7^. Notably, the molecular weight of HA influences immune cell activation: high molecular weight HA suppresses immune responses, while fragmented, low molecular weight HA promotes inflammation^10,11^. Proteoglycans including perlecan and decorin contribute to the ECM’s mechanical properties, regulate growth factor availability and influence cell proliferation and differentiation^7,10,12,13^. Together, these ECM components create a highly organized microenvironment that is critical for LN architecture and immunity.

Given the ECM’s role in immune homeostasis, its components are increasingly recognized as therapeutic targets across diseases^14,15^. In cancer, changes in ECM architecture drive tumor progression and indicate disease state^16^. Similarly, in autoimmune diseases like type 1 diabetes, collagen and laminin remodeling has been linked to immune cell infiltration and tissue destruction^11^. Multiple groups have shown that pancreatic LNs from individuals with recent-onset type 1 diabetes display altered ECM composition, including reduced germinal centers and fragmented HA, which promote immune activation and accelerate disease progression^17,18^. Our lab has also previously demonstrated that FRC networks in the pancreatic and skin-draining LNs of non-obese diabetic mice (NOD) display larger reticular pores than non-obese diabetic-resistant controls (NOR), which could promote diabetogenic T cell expansion in LNs^19^. Similar ECM changes have been observed in rheumatoid arthritis, whereby alterations in the LN stromal compartment have been linked to the onset of autoimmunity^20^. Despite these associations, the mechanisms linking ECM remodeling to immune dysfunction remain poorly understood, in part due to the lack of tissue-specific platforms to study stromal cell-ECM interactions in vitro and reliance on animal models.

Decellularized tissues are powerful tools for studying ECM microenvironments because they retain the native ECM structure, composition, and biochemical cues while removing confounding cellular elements^21^. Decellularized ECM scaffolds have been widely used to model tissue structure and function across organ systems, including heart, lung, pancreas, and kidney^22–27^. Decellularized LN (dLN) scaffolds have also been developed to induce antitumor immunity^28^ and, when reseeded with human adipose-derived stem cells, dLNs induced lymphangiogenesis^29^. However, most decellularized scaffolds are derived from whole organs and intended for transplantation or structural reconstruction, rather than for use in mechanistic, in vitro studies^30^. As a result, they often lack spatial control, limit uniform cell seeding, and are poorly suited for live imaging, co-culture studies and mechanistic analyses^27,31^. Several groups have established decellularization protocols that successfully preserve ECM components^28,32,33^. However, these protocols often yield dense tissue that is incompatible with high-resolution microscopy or downstream cell recovery. Moreover, the thickness of these decellularized scaffolds restricts oxygen and nutrient diffusion, leading to decreased cell viability and necrotic cores in the absence of vasculature^30^. Cells are often seeded onto tissue scaffolds via static seeding, in which cells are pipetted onto the scaffold surface and allowed to settle by gravity. Although widely used for its simplicity, this method often leads to poor cell penetration and uneven distribution, particularly in thick constructs produced by current LN decellularization techniques.

To address these limitations, we developed a method that combines vibratome sectioning with detergent decellularization to generate thin, dLN slices from mouse and human tissues. Vibratome sectioning is widely used to prepare viable tissue slices from organs such as the brain, pancreas, and lymphoid tissues for live-cell imaging, spatial transcriptomics, electrophysiology, and immune profiling^34–37^. For instance, pancreatic tissue slices have enabled spatial analysis of immune cell infiltration, islet architecture, and extracellular matrix remodeling, thereby enhancing our understanding of disease progression^38,39^. However, these applications often focus on short-term viability or imaging within preserved cell networks. In contrast, our platform applies vibratome slicing followed by decellularization, generating ECM-only scaffolds with defined geometry^36^ that are seeded with FRCs for long-term culture, live imaging, and eventual extraction for downstream analysis. This repurposing extends the use of the vibratome beyond live-tissue studies to create dLN slices for cell-dECM culture systems.

Precision-cut organ/tissue slices (PCOS/PTS) models retain native extracellular architecture and interactions^40^. However, cell culture is typically limited due to diffusion barriers and progressive tissue degeneration. In contrast, organotypic engineered models such as organoids or scaffold-based models can be cultured for longer periods with greater experimental control, but they lack native tissue organization and ECM cues^36^. Hence, our approach is placed at an intermediate position in which ECM cues are preserved while enabling uniform cell seeding, high-resolution imaging, and downstream applications not easily achievable in thick decellularized slices or conventional slice cultures.

After sectioning and decellularization, dLN sections were reseeded with FRCs. FRCs synthesize key LN ECM components, including collagen, fibronectin, and laminin, and regulate immune cell localization, survival, and antigen access^2,3,5,41^. We selected FRCs because of their role in LN ECM architecture and their responsiveness to matrix cues and composition^42^. Here, we demonstrate that our dLN sections support attachment, viability, and culture of mouse and human FRCs. Our work establishes the foundation for using our dLN sections in future functional studies of LN cell-ECM interactions in a native, lymphoid-like scaffold.

## Materials and methods

### Ethics statement

All animal experiments were conducted using protocols approved by the Institutional Animal Care and Use Committee of the University of Miami (IPROTO202400000648) and carried out in an AAALAC international-accredited facility. All animal procedures were performed in accordance with the relevant guidelines and regulations. Animals were housed under ambient conditions (temperatures of 20–23 °C with 40–60% humidity), allowed access to food and water ad libitum, and kept on a 12 h on/off light cycle. Our study is in accordance with ARRIVE guidelines.

Human LNs were obtained from cadaveric donors as discarded tissue designated for pancreatic islet isolation through the cGMP facility at the Diabetes Research Institute at the University of Miami. Human tissues were fully de-identified before use. The Institutional Review Board (IRB) of the University of Miami classified this study as IRB exempt. All methods were performed in accordance with relevant institutional guidelines and regulations. As our studies used fully de-identified, discarded tissue from donors who had already provided consent for donation prior to death, the requirement for further informed consent was waived by the University of Miami IRB.

### Lymph node harvesting

Skin-draining (cervical, inguinal, axillary, brachial, and mandibular) LNs were harvested from 12-week-old NOD (Jackson Laboratories, NOD/ShiLtJ, Strain #: 001,976) mice. Mice were euthanized using carbon dioxide followed by cervical dislocation, as per approved protocols. At the time of euthanasia, all female mice weighed a minimum of 24 g, and all male mice weighed at least 30 g, which are within the healthy physiological range for mice at this age. For each experiment, three to five male and female mice were used as biological replicates per group. LNs from each mouse were pooled to minimize variability between individual LNs and to increase total tissue yield. Male and female mice were randomly selected for each experiment to control for potential sex-related differences.

Dissected human LNs obtained from cadaveric donors were washed in Phosphate Buffered Saline (PBS, Gibco, cat# 10010023) or placed in MACS® Tissue Storage Solution (Milteny, cat# 130–100-008). All excess fat and connective tissue were carefully removed using dissecting scissors or a scalpel under sterile conditions.

### Lymph node sectioning

Harvested LNs were embedded in varying percentages of agarose (3, 6, and 8%; Promega, cat# V2111) and sectioned using a vibratome (Leica, VT1200 S). At least nine pooled skin-draining LNs were sectioned per mouse and three to five mice per group were used as biological replicates. Slicing parameters, including section thickness (120–200 μm) and blade velocity (1.0 or 1.5 mm/s), were systematically tested to maximize recovery rate, defined as the percentage of intact slices retrieved per trial. The sectioning buffer consisted of chilled PBS, and all procedures were performed on ice to maintain tissue integrity. The final sectioning conditions selected for downstream validation studies were determined based on the highest observed recovery rate: 6% agarose, 1 mm/s blade velocity, and 200 μm section thickness. These settings correspond to the conditions in Trial 8 (Supplementary Table 1). After carefully removing the surrounding adipose tissue, agarose was heated until liquefied and subsequently cooled to 37–40 °C. The lymph nodes were then fully embedded in the molten agarose, ensuring complete coverage. Once the agarose had solidified, the embedded tissue was trimmed using a biopsy punch, mounted onto a vibratome specimen disc, and sectioned using a vibratome.

### Decellularization of whole lymph nodes and lymph node sections

The LN decellularization protocols were optimized and adapted from previously reported methods^22,32^, including changes in detergent delivery and incubation timing. These adjustments were necessary to efficiently decellularize vibratome-cut LN slices as these differ from whole organs in geometry, surface area, and diffusion dynamics.

For Protocol A, adapted from Cuzzone and colleagues^32^, LN fragments were placed in 0.075% Sodium Dodecyl Sulfate solution (SDS, ThermoFisher Scientific, cat# 28365) prepared in distilled water and agitated at 150 rpm for 16 h at 37 °C to remove cellular components. Following this step, the samples were washed thoroughly with PBS in fresh tubes five times to eliminate residual detergent and debris.

Due to the amount of time required for this protocol, Protocol B, adapted from Citro et al^22^, was evaluated. Varying concentrations of SDS were tested for decellularization (0.1, 0.2, 0.5 and 1% SDS). Whole LNs were injected with SDS using a syringe until its pink color transitioned to a transparent appearance. The tissue was then incubated in fresh 1 mL volumes of SDS solution and replaced every 30 min for two hours. For vibratome-obtained LN sections, 0.1% SDS was applied without injection. After SDS treatment, both whole LNs and LN sections were thoroughly washed with deionized water to remove residual SDS and incubated in 1 mL of DI water for 20 min. To improve lipid removal, samples were subsequently incubated in 1 mL of 1% Triton X-100 (Sigma, cat# 9036–19-5) for 20 min. Following this step, samples were washed five times in sterile PBS. For downstream cell culture applications, dLN sections were incubated overnight at 4 °C in 1 mL of a 1% Antibiotic–Antimycotic solution (Gibco, cat# 15240062) diluted in PBS to prevent microbial contamination prior to reseeding.

### DNA quantification assay

DNA concentration (ng/mL) in LNs and LN sections before and after decellularization was assessed using the Quant-iT™ PicoGreen™ dsDNA Assay (ThermoFisher Scientific, cat# P7589) following the manufacturer’s protocol. This assay is widely used for evaluating DNA removal in decellularized tissues due to its high sensitivity, capable of detecting double-stranded DNA levels as low as 25 pg/mL^43,44^.

Briefly, whole LNs and LN sections were lysed in 10 mM TE Buffer (Tris–HCl, and1 mM EDTA) and frozen at -80 °C for at least 1 h. Upon thawing, lysates were vortexed and centrifuged at 4 °C for 20 min. The resulting supernatant was combined with the Quant-iT™ PicoGreen™ dsDNA Reagent, incubated at room temperature for 5 min (protected from light), and fluorescence was measured using a microplate reader (excitation: 480 nm, emission: 520 nm).

### Extracellular matrix quantification assays

Glycosaminoglycan (GAG) and total collagen content were measured in LNs and LN sections before and after decellularization using colorimetric assays, per manufacturer protocols.

#### GAG quantification

GAG content was assessed using the Total Glycosaminoglycans Assay Kit (Abcam, cat# ab289842). Briefly, samples were homogenized in GAG buffer, centrifuged at 4 °C for 20 min, and the supernatant was collected. The GAG detection reagent was added and incubated for 2 min at room temperature. All reagents used were provided in the kit, including GAG assay buffer, probe, and standards. Absorbance was read at 400 nm using a plate reader. Results were normalized to wet tissue weight and conducted in triplicate.

#### Collagen quantification

Collagen was measured using the Total Collagen Assay Kit (Abcam, cat# ab222942). For each sample, 10 mg of tissue was homogenized in 100 μL of distilled water, then hydrolyzed in concentrated NaOH at 120 °C for 1 h. Hydrolysates were cooled on ice, neutralized with concentrated HCl, and centrifuged at 10,000 × g for 5 min.

The supernatant was transferred to wells, evaporated at 65 °C, and combined with the oxidation solution. After 20 min, developer and DMAB reagents were sequentially added, followed by incubation at 65 °C for 5 and 45 min, respectively. Absorbance was measured at 560 nm using a microplate reader. All assays were performed in triplicate and normalized to wet tissue weight.

### Immunofluorescence studies

LN sections (before and after decellularization) were stained and imaged using a confocal microscope (Stellaris, Leica Instruments) to assess extracellular matrix preservation and cell compatibility. Antibodies targeting alpha-smooth muscle actin (α-SMA)-Cy3 (1:200, Sigma, cat# C6198), Pan-Laminin (1:250, Abcam, cat# ab7463), Collagen IV-AF488 (1:150, Abcam, cat# ab309502), Polyclonal Collagen I (1:200, Invitrogen, cat# PA5-95137) were used to visualize ECM proteins. Cytoskeletal and nuclear staining included F-actin Phalloidin-AF546 (1:400, Invitrogen, cat# A22283), F-actin Phalloidin-AF647 (1:100, Invitrogen, cat# A22287), Vimentin-AF647 (1:150, Cell Signaling, cat# 9856) and DAPI (1:10,000, Invitrogen, cat# D21490).

Stained sections were mounted in ProLong Gold Antifade Mountant (Invitrogen, cat# P36930) and imaged with consistent laser settings across conditions. At least 3 biological replicates were imaged per group, with representative images shown. All image analysis was performed using LAS X Life Science Microscope Software (Leica Instruments) with consistent thresholding across samples. Negative controls without primary antibody were used to assess non-specific secondary antibody binding.

### Atomic force microscopy

Atomic Force Microscopy (AFM) was used to compare the elasticity (Young’s Modulus) of the LN sections before and after decellularization to assess how detergent treatment affected ECM mechanical properties^45^. Sections were immobilized on 35-mm tissue culture dishes using a 0.5% agarose base. After plating 50 μL of agarose gel onto pre-warmed dishes (25 °C), sections were placed into the gel prior to solidification. Once gelled, plates were filled with PBS to maintain hydration during measurements.

AFM measurements were performed using a custom-built AFM system optimized for soft tissue characterization^46^. A spherical indenter tip (2 μm diameter, k = 0.1 N/m; Novascan Technologies, USA) mounted on a piezoelectric actuator (P-841.40, Physik Instrumente, Germany) was used. Indentation parameters included a 15 μm/s approach and retraction speed and a maximum indentation force of ~ 12 nN (1 V). Each sample (7–10 LN sections per group, derived from 3 biological replicates per group) was measured at three distinct regions, and each region was indented 10 times.

Resulting force-indentation curves were analyzed in MATLAB and fit to the Hertz model for a spherical indenter:

$$F = \frac{4E\sqrt R }{{3\left( {1 - v^{2} } \right)}}D^{3/2}$$

where F is the force in Newtons, v is the Poisson’s ratio (0.49), D is the indentation (m), R is the radius of the indenter (m), and E is the Young’s modulus of elasticity (Pa). Outliers were omitted using the interquartile range method, whereby any value 1.5 times above the third quartile or 1.5 times below the first quartile was considered an outlier.

### Stromal cell seeding and culture in decellularized lymph node sections

Murine and human FRCs were isolated from skin-draining LNs of 12-week-old female NOD or C57BL/6 J (Jackson Laboratories, Strain #: 000664) mice, or cadaveric human donors using established protocols^47^. Isolated cells were expanded (passage 25 for NOD and B6 murine FRCs and passage 14 for human FRCs) and seeded onto dLN sections. dLN sections were sterilized under ultraviolet light for 30 min before cells were seeded.

NOD FRCs were resuspended in 5 μL of FRC culture media at concentrations of 30,000, 50,000, or 100,000 cells per section and allowed to attach for 1 h before the wells were supplemented with an additional 200 μL of media. Seeding densities ranging from 6,250 to 100,000 cells/well were tested to generate a standard curve correlating bioluminescence with viable cell number. Human FRCs were seeded at a concentration of 30,000 FRCs per human dLN section.

Longitudinal viability of seeded FRCs was assessed for up to 28 days using the RealTime-Glo™ MT Cell Viability Assay (Promega, cat# G9711) following the manufacturer’s protocol. Briefly, 200 μL of the reagent/media mix was added to each well and incubated at 37 °C for 1 h. Bioluminescence was recorded for 1 s using a microplate reader. Each condition was tested in biological triplicates.

### Stromal cell extraction from decellularized LN sections

NOD FRCs were extracted from dLN sections using various enzymatic protocols (Supplementary Table 2) to determine the most effective method for recovering viable cells. The goal was to identify a condition that maximized both total and live cell recovery. Enzymatic reagents included Trypsin–EDTA (Gibco, 25200056) and a previously reported Enzyme Mix^47^ were used as enzymatic reagents to extract FRCs from the seeded, dLN sections.

Protocols varied in terms of reagent volume, mechanical disruption, incubation time, and vortexing. The most favorable protocol (Method 5, Supplementary Table 2) involved incubating dLN sections with 1 mL of Enzyme Mix for 3 min while applying gentle mechanical disruption using a low-speed vortex for 5 s. This condition resulted in the highest total cell recovery (210,000 cells) and viability (66%). This protocol was selected for subsequent studies based on its high total yield and viability, making it the most favorable. For extraction, four dLN sections were incubated in 1 mL of ice-cold enzyme mix. Immediately upon addition, sections were mechanically disrupted by gentle pipetting until visibly dissociated. The mixture was incubated at 37 °C for 3 min, followed by vortexing at low speed for 5 s. Remaining tissue fragments were allowed to settle, and the supernatant containing released cells was transferred to 5 mL of sterile FACS buffer (PBS with 2 mM EDTA; Gibco, cat# 15575–038) containing 2% Fetal Bovine Serum (Gemini Bio, cat# 100–106, Lot A324002). Samples were centrifuged at 25 °C, and the resulting cell pellet was resuspended in fresh media for counting and downstream applications.

To validate whether culturing FRCs in dLN sections could enable cell analyses, NOD or B6 FRCs were extracted from dLN sections, extracted FRCs were analyzed on a Cytek Aurora flow cytometer (Cytek Biosciences, USA) and processed using Kaluza software (Beckman Coulter, USA). For staining FRCs, cells were plated in a 96-well V-bottom plate, washed twice with PBS, and incubated with Live/Dead-Blue (Invitrogen, cat# L23105, 1:1000) for 20 min. Cells were then washed with FACS buffer (PBS, 2 mM EDTA; Gibco, 15575–038) containing 2% Fetal Bovine Serum (Gemini Bio, cat# 100–106, Lot A324002), and stained with antibodies against FRC phenotypic markers: gp38 (Invitrogen, cat# 12–5381-82, PE, 1:100) and PDGFR-α (BioLegend, cat# 135922, PE-Dazzle594, 1:160). After staining, cells were washed and resuspended in FACS buffer prior to flow cytometry assessment. Controls included unlabeled cells, Live/Dead-Blue stained cells, and single antibody labeled UltraComp eBeads Plus Compensation Beads (Invitrogen, cat# 01–3333-42). Gp38 and PDGFR-α expression in NOD or B6-derived FRCs were calculated from live singlets.

### FRC and T cell co-culture

To assess the potential of using our dLN sections as a co-culture platform for antigen-presenting cells such as FRCs with T cells, an in vitro co-culture assay was adapted based on established protocols^19,42^. Briefly, 30,000 NOD-derived FRCs were seeded onto NOD dLN sections in 48-well plates, cultured for 11 days, and then pre-conditioned with 10 ng/mL interferon-gamma (IFNγ) for 3 days. During the final 24 h of IFN-γ treatment, cells were additionally pulsed with 0.011 μg/mL of IGRP_206-214_ peptide (vylktnvfl) to enable antigen presentation by FRCs to antigen-specific CD8^+^ T cells from NY8.3 mice^48^. these NY8.3CDd8^+^ tTcells specifically recognize the IGRP_206-214_ peptide presented on NOD-derived MHC-I by antigen presenting cells. Antigen-specific (IGRP-reactive) naïve CD8⁺ t cells were isolated from spleens of 12-week-old NY8.3 female mice using magnetic-activated cell sorting (STEMCELL Technologies, cat# 19853). Prior to co-culture, T cells were labeled with CellTrace Violet (Invitrogen, cat# C34557) to assess proliferation. Following FRC pre-conditioning, 100,000 NY8.3 CD8⁺ T cells were added to each dLN section with NOD FRCs and co-cultured for 3 days. Negative controls included FRC-seeded dLN sections not pulsed with antigen. As a positive control for T cell activation and proliferation, 100,000 NY8.3 CD8^+^ T cells were cultured with αCD3/CD28 Dynabeads (Gibco, cat# 11453D), following the manufacturer’s instructions. After co-culture, T cells were collected and stained following the same protocol described for FRC staining. T cell antibodies for flow cytometry included CD44 (BioLegend, cat# 103047, Brilliant Violet 605, 1:500), CD8 (BD Horizon, cat# 566409, BB700, 1:400), and CD3 (BioLegend, cat# 100272, PE/Fire 700, 1:320). Percent CD44 expression and CellTrace Violet dilution (indicative of proliferation) were calculated from live, CD3^+^, CD8^+^ singlets.

### Data analysis

Data is represented as mean ± standard deviation or standard error of the mean. Data was graphed using GraphPad Prism version 10.0.0 for Windows (GraphPad Software, USA) and p-values were determined by unpaired Student’s t-test, and one or two-way ANOVA, followed by Tukey’s multiple comparison test with Šidák correction where appropriate. (*p < 0.05; **p < 0.01; ***p < 0.001; ****p < 0.0001; ns, not significant, p > 0.05).

## Results

**Fig. 1:**
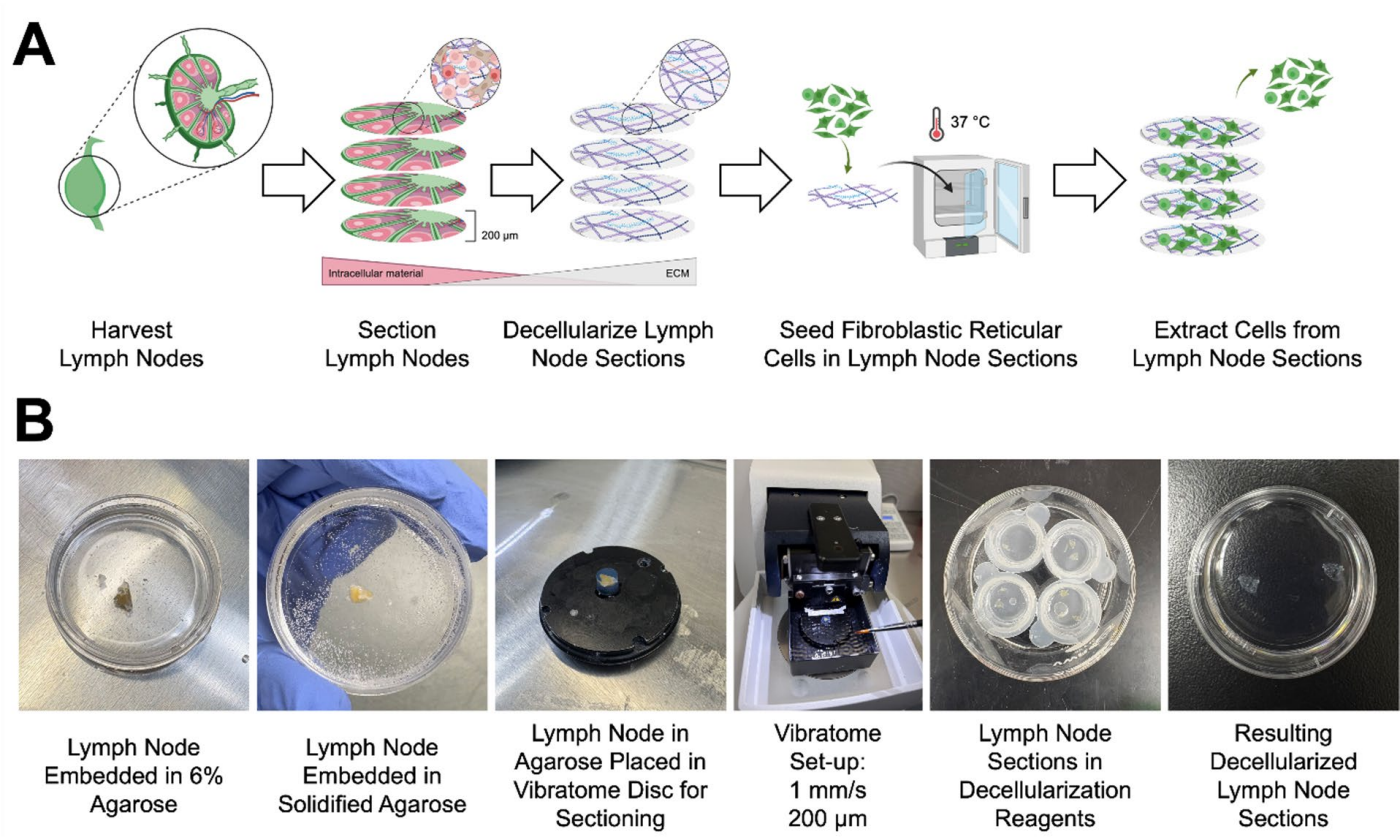
Lymph node sectioning and decellularization schematic and workflow. **(A)** Murine and human lymph nodes were harvested, embedded in agarose, sectioned into 200 μm slices using a vibratome, and decellularized with SDS and Triton-X. The dLN sections were then seeded with FRCs, cultured for 1–28 days, and subsequently harvested using an enzymatic digestion mix for further downstream analysis. (**B**) Dissected lymph nodes were embedded in 6% agarose and allowed to solidify. Once set, samples are cored using a 6-mm (mouse) or 8-mm (human) biopsy punch and mounted into the vibratome disc. Lymph nodes were sectioned into 200 μm sections using a vibratome. The resulting sections were then decellularized.

A systematic workflow was established to produce dLN sections for FRC culture and recovery for downstream cell analysis (Fig. 1A). This process began with harvesting and trimming excess adipose tissue from skin-draining LNs from 12-week-old NOD or B6 mice and pancreatic LNs from healthy human donors. LNs were then embedded in agarose and sectioned using a vibratome (Fig. 1B). Various sectioning parameters were tested, including thickness, velocities, and agarose concentrations (Supplementary Table 1). Among the eight conditions tested, Trial 8 (6% agarose embedding, 1 mm/s cutting speed, and 200 μm thickness) produced the highest recovery rate, with 75% of sections remaining intact. Trial 7 (8% agarose and same cutting parameters) yielded an equivalent recovery rate. However, 6% agarose was selected for the final protocol to reduce agarose matrix density and facilitate downstream diffusion of decellularization reagents and cell media. Lower agarose concentration was also favored to minimize potential interference with imaging and cell extraction processes.

### Decellularization protocols remove DNA from mouse lymph nodes and vibratome sections

**Fig. 2:**
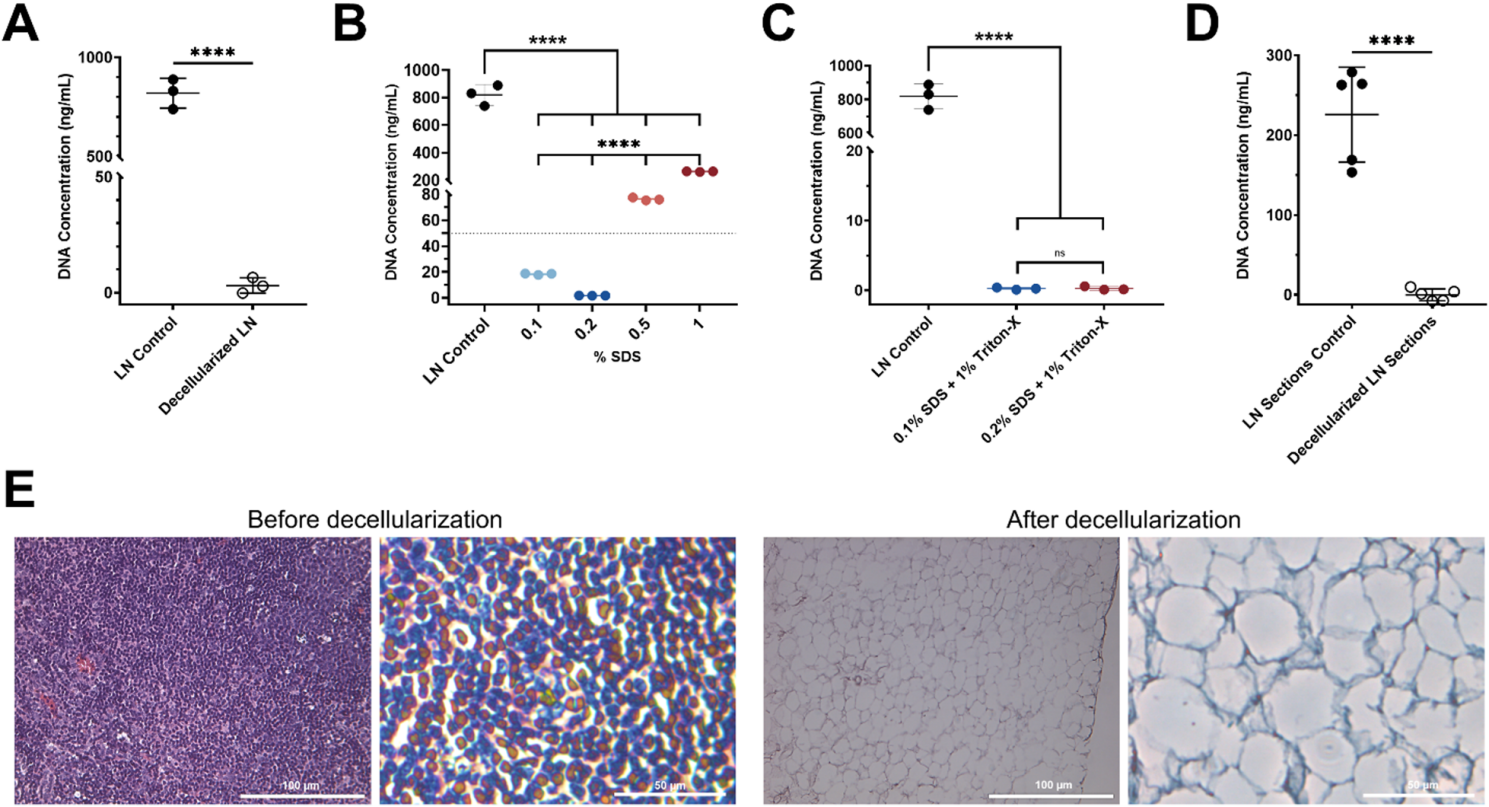
DNA quantification and histological assessment of control and decellularized lymph nodes. (**A**) DNA concentration in control and decellularized lymph nodes using Protocol A (0.075% SDS; duration: 16 h) (n = 3). (**B**) Effect of SDS concentration on DNA concentration in decellularized lymph node sections (n = 3). DNA concentrations are significantly different among all SDS treatments and relative to the LN control. (**C**) Effect of 1% Triton-X addition following SDS treatment (n = 3). (**D**) DNA concentration in control and decellularized lymph node sections using Protocol B (0.1% SDS and 1% Triton-X; duration: 3 h) (n = 5). (**E**) Light images of H&E-stained control and decellularized lymph nodes using Protocol B (scale bar = 100 μm, left; 50 μm, right). Data represented as mean ± standard deviation. P-values were determined by unpaired, student’s t-test or ordinary one-way ANOVA followed by Tukey’s multiple comparison test. (****p < 0.0001; ns, not significant, p > 0.05).

To evaluate the efficacy of the decellularization protocols, we quantified DNA content in murine LNs before and after decellularization using the Quant-iT™ PicoGreen™ dsDNA assay. Protocol A (0.075% SDS, 16 h) significantly reduced DNA levels compared to untreated controls (Fig. 2A) but required prolonged incubation. To shorten the protocol, we tested a range of SDS concentrations (0.1–1%) over a 3-h protocol (Protocol B, Fig. 2B). DNA concentrations differed significantly among all SDS treatments and compared with the control (p < 0.0001), with 0.2% SDS resulting in the greatest DNA removal, followed by 0.1% SDS. This cutoff is considered the gold standard for complete decellularization to minimize immunogenicity of residual nucleic acids^21,49^. Higher SDS concentrations (0.5% and 1%) increased residual DNA content. Because prolonged SDS exposure and higher concentrations can compromise ECM and LN section integrity^50,51^, we incorporated 1% Triton X-100 as a secondary detergent to support effective decellularization while preserving tissue structure (Fig. 2C). Triton X-100 significantly reduced DNA levels across SDS conditions compared to the LN control (p < 0.0001), and no significant difference was observed between 0.1% and 0.2% SDS when combined with Triton X-100. However, treatment of dLN sections with 0.2% SDS and 1% Triton X-100 compromised the integrity of the thin sections. Therefore, 0.1% SDS followed by 1% Triton X-100 was selected for LN section decellularization. This protocol resulted in complete decellularization, as evidenced by negligible DNA levels compared to control LN sections (Fig. 2D). A representative example of lymph node morphology before and after decellularization is shown in Supplemental Fig. 1, demonstrating that the decellularization protocol does not compromise LN section integrity. Hematoxylin and eosin (H&E) staining of LN sections before and after decellularization further confirmed complete removal of nuclear and cytoplasmic cellular material, while retaining ECM architecture (Fig. 2E). Control LNs displayed densely packed nuclei, while dLNs showed translucent matrices devoid of visible cells. Overall, these results confirm the visual and quantitative efficacy of the decellularization process.

### Decellularization protocol retains extracellular matrix components and mechanical properties

**Fig. 3:**
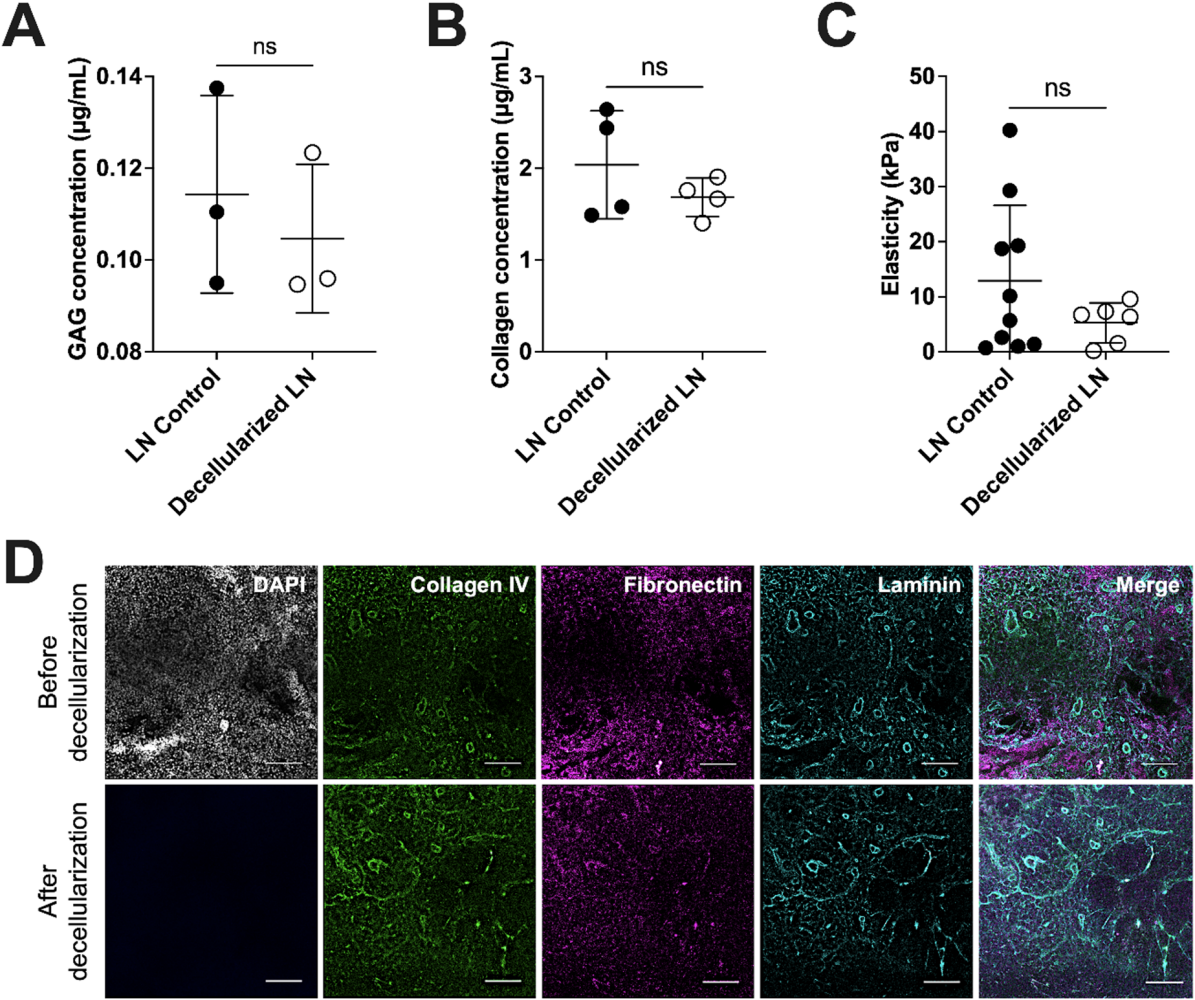
Decellularized lymph node characterization and ECM protein preservation. (**A**) Glycosaminoglycan (GAG) concentration in control and decellularized lymph nodes (n = 3). GAG concentration was measured using Abcam’s colorimetric Total Glycosaminoglycans kit (ab289842). (**B**) Total collagen concentration in control and decellularized lymph nodes (n = 4). Collagen concentration was measured using Abcam’s Total Collagen Assay Kit (Perchlorate-Free) (ab222942). (**C**) Elasticity measured by Atomic Force Microscopy in control and decellularized lymph node sections (n = 6–10). (**D**) Representative immunofluorescence images of control and decellularized lymph node sections stained for DAPI (Nuclei, gray), and ECM proteins Collagen IV (green), Fibronectin (magenta), and Pan-Laminin (cyan) (scale bar = 100 μm). Data represented as mean ± standard deviation. P-values were determined by unpaired, student’s t-test. (ns, not significant, p > 0.05).

To assess whether ECM content and mechanical properties remained present following decellularization, we performed biochemical assays and immunofluorescence imaging (Fig. 3). Quantitative analysis revealed that GAG (Fig. 3A) and total collagen (Fig. 3B) concentrations were not significantly different between control and dLN sections, indicating that the decellularization protocol preserved these ECM components. Given the importance of ECM mechanics in regulating stromal cell behavior^52^, we measured the elasticity (Young’s modulus) of dLN sections via AFM (Fig. 3C). No significant differences were observed in elasticity between control and dLN sections. However, control LN sections showed greater variability in elasticity values, consistent with its higher cellular content and structural heterogeneity prior to decellularization.

Immunofluorescence staining and confocal imaging further confirmed the presence of ECM proteins, including collagen IV, fibronectin, and laminin (Fig. 3D). DAPI staining showed complete removal of cellular nuclei post-decellularization, while ECM markers remained visible and spatially organized. Merged images suggest that the ECM network remained largely continuous after decellularization, supporting its potential suitability for subsequent FRC attachment and culture.

These findings demonstrate that our protocol efficiently removed cellular material while preserving ECM mechanical properties and the presence of ECM proteins in both native and decellularized LNs, thereby supporting subsequent FRC seeding and culture in dLNs.

### Decellularized lymph node sections enable murine FRC culture, characterization, and co-culture with T cells

**Fig. 4:**
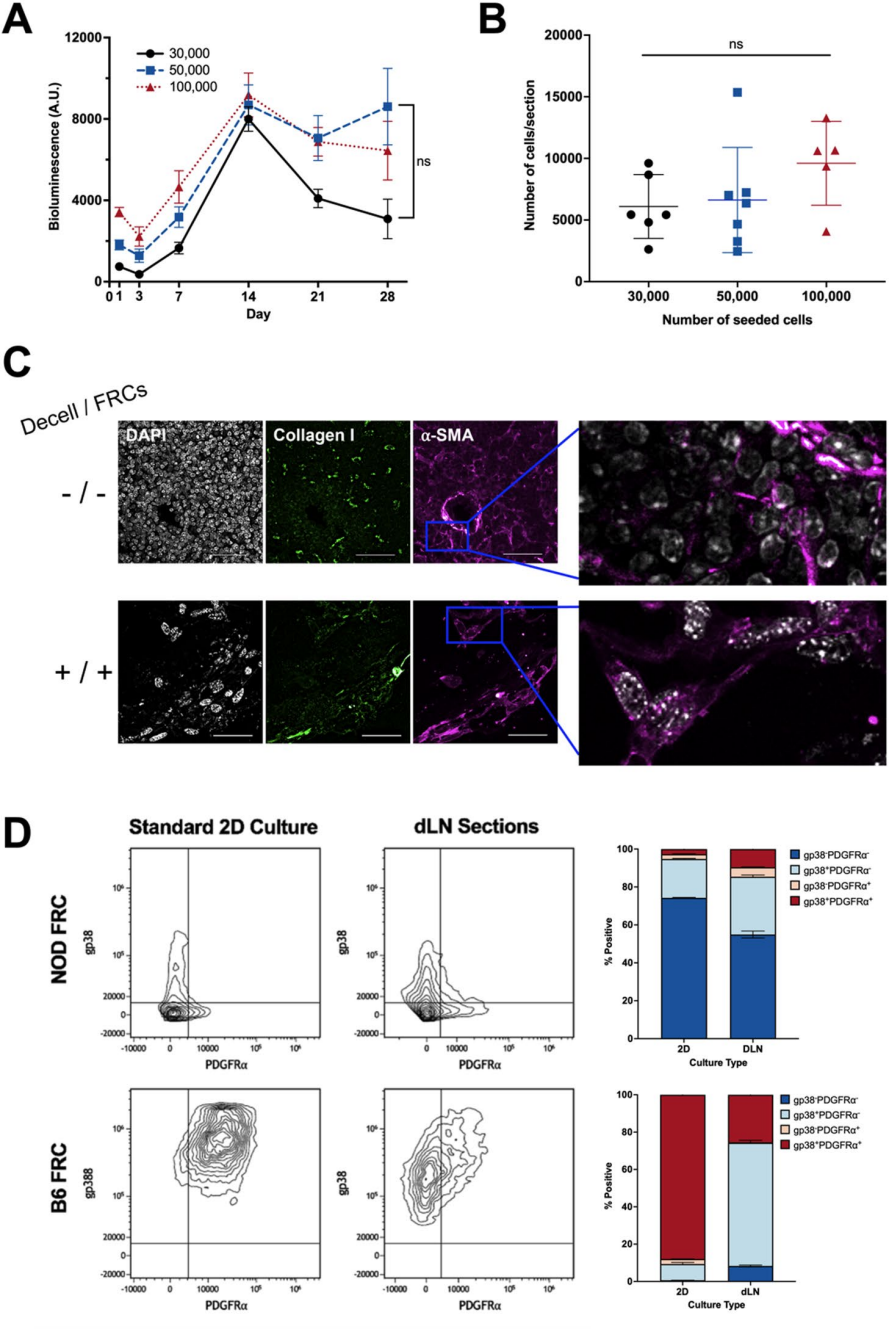
FRC culture on decellularized lymph node sections. Murine NOD-derived FRCs were seeded at densities of 30,000, 50,000, and 100,000 cells per 200 μm dLN section and cultured for up to 28 days in vitro. (**A**) Bioluminescence longitudinal assessment of cell viability via RealTime-Glo™ assay for 1–28 days (n = 5 dLN sections seeded with FRCs per group). (**B**) NOD FRC retention in dLN sections as the number of viable FRCs per section, quantified at day 7 using a standard curve of known FRC numbers and bioluminescence (n = 5 sections per group). (**C**) Representative immunofluorescence images of (top) control non-decellularized LN section (-/-) and (bottom) dLN sections re-seeded with NOD-derived FRCs (+ / +) and cultured for 14 days, stained for DAPI (Nuclei, gray), ECM marker (collagen I: green), and myofibroblastic cell marker α-SMA (magenta), including DAPI and α-SMA merged zoom (right). Scale bar = 50 μm. Decell = Decellularized; FRCs = Seeded with FRCs. (**D**) Representative flow cytometry plots of NOD and B6-derived FRCs isolated from NOD and B6 dLN sections, respectively, after 14 days of culture (30,000 FRCs initially seeded, n = 3 biological replicates with 3 pooled sections with passage-matched FRCs per replicate), and % expression of FRC phenotypic markers gp38 (PE) and fibroblast marker PDGFR⍺ (PE-Dazzle594). Cells gated on live singlets. p < 0.0001 between 2D and dLN cultures for all four groups (gp38^-^PDGFRα^-^, gp38^-^PDGFRα^+^, gp38^+^PDGFRα^-^, gp38^+^PDGFRα^+^) in NOD- and B6-derived FRCs. Data represented as mean ± standard deviation. P-values were determined by two-way or ordinary one-way ANOVA followed by Tukey and Šidák correction or unpaired Student’s t-test. (ns, not significant, p > 0.05).

To evaluate whether dLN sections could support LN-derived stromal cells, murine FRCs, a type of stromal cell, were seeded at densities of 30,000, 50,000, and 100,000 FRCs per dLN section and cultured for 28 days in vitro using RealTime-Glo™ assay. FRC bioluminescence steadily increased over the first 14 days of culture across all groups, suggesting that FRCs proliferated within the dLN sections. After day 14, bioluminescence remained constant or decreased slightly, with no statistically significant differences among seeding densities (Fig. 4A). These results suggest that our dLN sections can support FRC culture, and microscope images show that dLN sections with FRCs remain intact during culture (Figure S2).

Using a standard curve correlating FRC number and luminescence signal, we determined that each 200-μm dLN section could support a maximum of 25,000 viable cells (Fig. 4B). On day 7, comparable FRC viability was observed across all initial seeding densities, suggesting that lower cell seeding densities may be sufficient for FRC attachment and proliferation within the matrix. Based on these results, 30,000 FRC were seeded per section for subsequent experiments, balancing seeding efficiency with the available area for FRC growth within the dLN sections. Since day 14 corresponded to peak bioluminescence levels across all conditions, this timepoint was selected for subsequent immunofluorescence and flow cytometry analyses.

Immunofluorescence staining and confocal imaging confirmed the expression of fibroblast (α-SMA, vimentin) and ECM (collagen I) proteins in FRCs seeded and cultured within dLN sections for 14 days (Figure 4C, S3). These results indicate that our dLN sections can allow for the visualization of fibroblast phenotypic markers after 14 days. Specifically, magnified immunofluorescent images of α-SMA and nuclei show the morphology of native LN cells ( -/-) compared to reseeded cells in dLN sections (+/+). Native lymph node sections contain abundant cellular components, whereas reseeded scaffolds show fewer cells overall but exhibit similar spindle- or elongated-like αSMA⁺ structures, consistent with fibroblastic morphology.

To facilitate downstream analyses, we evaluated enzymatic digestion protocols for extracting viable FRCs from dLN sections. Five protocols were tested by varying enzymatic composition, incubation time, and degree of mechanical disruption (Supplementary Table 2). Method 5, consisting of 1 mL of enzyme mix, gentle pipette disruption, a 3-min incubation, and a 5-s low-speed vortex, yielded the highest total and viable cell counts. This method was therefore selected for the extraction of NOD- or B6-derived FRCs from dLN sections after culture for 14 days. Following cell extraction, cell phenotype was characterized using flow cytometry. Recovered FRCs formed viable single-cell suspensions expressing gp38 and PDGFRα (Fig. 4D). FRCs are defined by their expression of gp38 and the absence of hematopoietic (CD45) and endothelial (CD31) markers. However, previous studies in human samples have shown that gp38 expression can vary considerably in 2D culture, even when identical isolation protocols are used^53^. To account for this variability, FRCs derived from two different mouse strains (NOD and B6) were compared. Consistent with our previous findings^42^, NOD FRCs displayed heterogeneous gp38 expression at baseline. This comparison enabled us to evaluate whether our dLN sections could capture biologically relevant changes in stromal phenotypes over time. After 14 days in culture in dLN sections or in standard two-dimensional culture flasks, FRC phenotypic markers gp38 and PDGFRα varied significantly depending on both the origin of the cells and the culture substrate. Significant differences were observed across the four groups when comparing % positive cells (gp38^-^PDGFRα^-^, gp38^-^PDGFRα^+^, gp38^+^PDGFRα^-^, gp38^+^PDGFRα^+^) between 2D and dLN cultures for NOD- and B6-derived FRCs, respectively (p < 0.0001 for all groups, Fig. 4D). NOD-derived FRCs, which initially exhibited low gp38 expression, showed a modest increase in gp38 when cultured within NOD dLN sections. In contrast, B6-derived FRCs, which expressed high gp38 levels at baseline, maintained gp38 expression overall, with the gp38^-^ subpopulation no longer detectable. PDGFRα, a fibroblast marker involved in fibroblast proliferation^54,55^, wound healing, and the fibroblast-to-myofibroblast transition^56^, was reduced in B6 FRCs after culture, suggesting context-dependent modulation of fibroblast-associated markers within the dLN microenvironment.

**Fig. 5:**
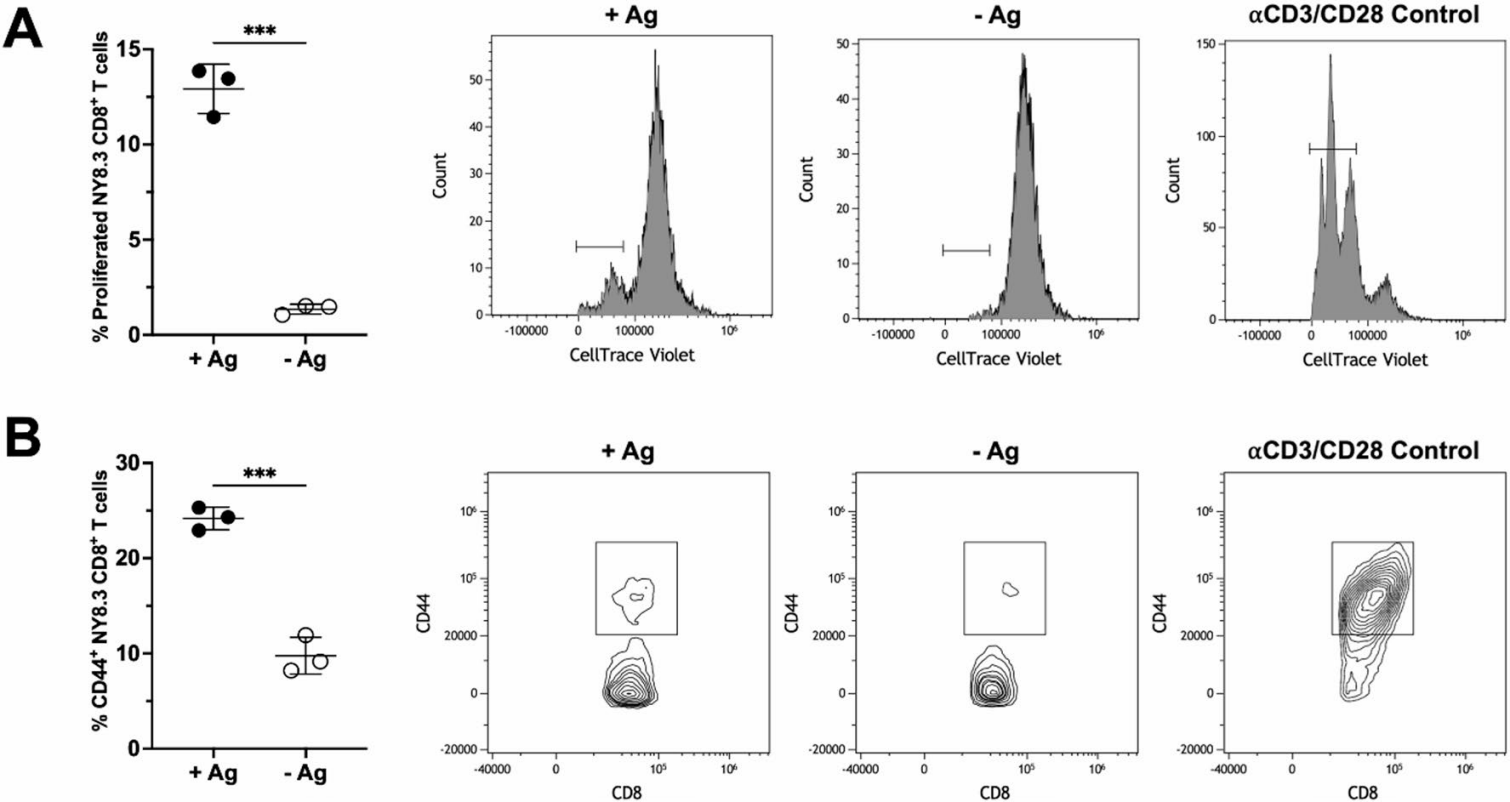
Proliferation and CD44 expression of Antigen-specific T cells in 3-day co-culture with NOD FRCs in dLN sections. Representative flow cytometry plots and quantification of NY8.3, Antigen-specific CD8^+^ T cell (**A**) proliferation (measured by CellTrace dilution) and (**B**) CD44 expression (indicative of antigen experience), after interaction with FRCs with (+ Ag) or without (-Ag) IGRP Ag in co-culture with NY8.3^+^ CD8^+^ T cells (n = 3). Cells gated on live, CD3^+^ singlets. Co-culture flow cytometry controls include co-cultures without the addition of soluble Ag (negative control) and αCD3/CD28 Dynabeads (positive control for flow cytometry gating). Data represented as mean ± standard deviation. P-values were determined by unpaired, student’s t-test. (***p < 0.001).

Next, to evaluate whether dLN sections could serve as a physiologically relevant platform for FRC-T cell co-culture studies, enabling interactions between these cell types, we co-cultured antigen-specific NY8.3 CD8⁺ T cells with NOD-derived FRCs for 3 days (Fig. 5), using established protocols^19,42^. Antigen-specific T cells co-cultured with antigen-pulsed (+ Ag) FRCs proliferated (Fig. 5A) and showed upregulation of CD44 expression, indicative of antigen experience (Fig. 5B). In contrast, T cells co-cultured with FRCs that were not pulsed with antigen (-Ag) had minimal activation or proliferation. These results suggest that FRCs seeded in dLN sections can effectively present antigen and promote activation and proliferation of antigen-specific T cells.

### Decellularization protocols apply to human LNs and support human FRC culture

To assess the translational potential of our decellularization and sectioning methods, we extended our protocols to human LNs obtained from multiple donors without any exclusion criteria. We aimed to evaluate the robustness of our approach across a diverse donor pool by assessing how human LNs could be consistently decellularized, reseeded with human FRCs, and stained for ECM proteins via immunofluorescence.

**Fig. 6:**
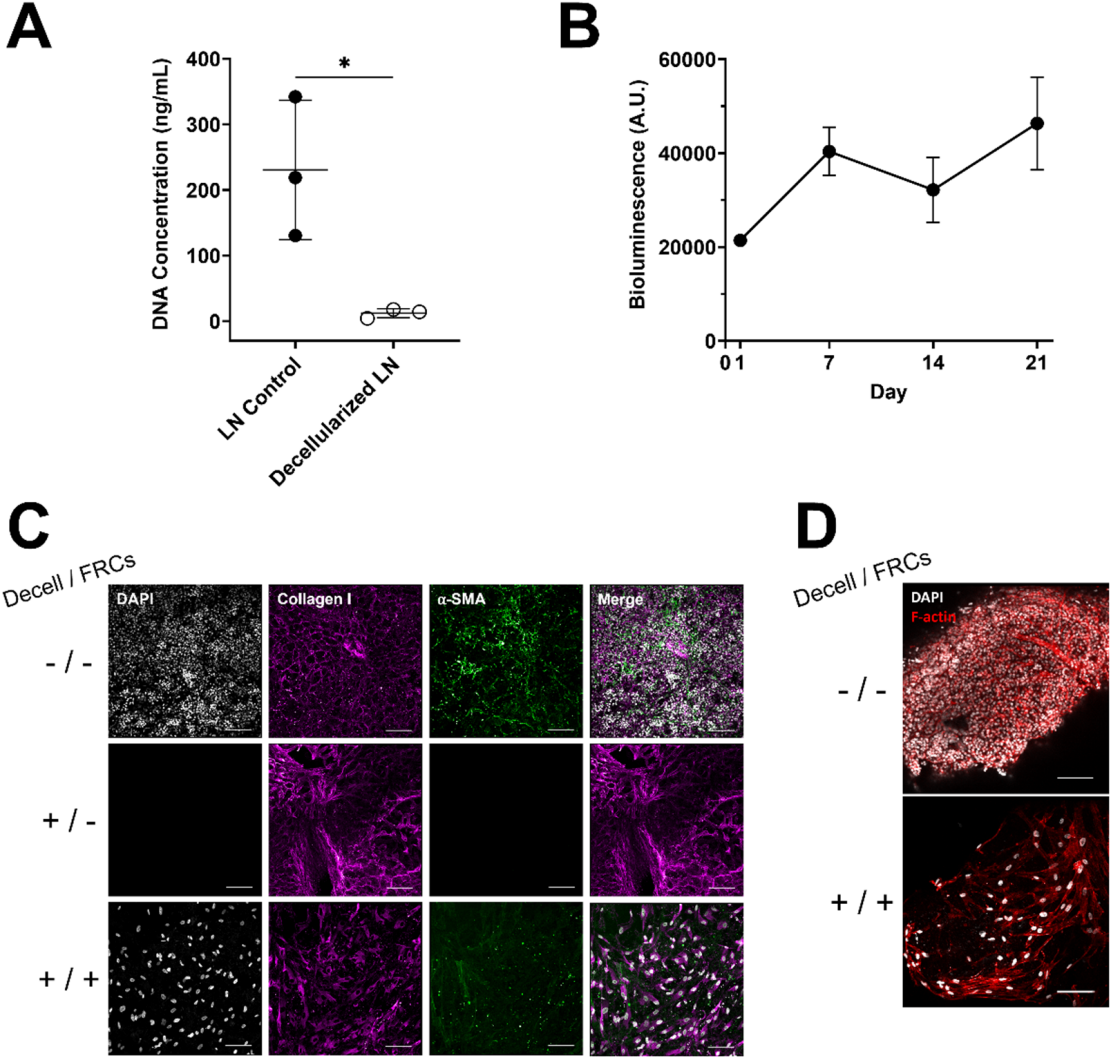
Human validation of decellularized lymph node sections. Human lymph nodes were sectioned (-/-), decellularized (+ /-), and seeded (+ / +) with human FRCs. (**A**) DNA concentration in control and decellularized human lymph nodes (n = 3 human donors). (**B**) Bioluminescence longitudinal assessment of human FRC viability via RealTime-Glo™ assay for 21 days (n = 5 human dLN sections seeded with 30,000 human FRCs per dLN section). (**C**) Representative immunofluorescence confocal images of control non-decellularized LN section (-/-), dLN sections before cell re-seeding (+ /-), and dLN sections seeded with human FRCs (+ / +) and cultured for 14 days stained for DAPI (Nuclei, gray), Collagen I (magenta), and α-SMA (green). Scale bar = 100 μm. Decell = Decellularized; FRCs = Seeded with FRCs. (**D**) Representative immunofluorescence confocal images of control non-decellularized LN sections (-/-) and dLN sections seeded with human FRCs (+ / +) and cultured for 14 days stained for DAPI (Nuclei, gray) and F-actin (red). Scale bar = 100 μm. Decell = Decellularized; FRCs = Seeded with FRCs. Data represented as mean ± standard deviation. P-values were determined by unpaired, student’s t-test (*p < 0.01).

Quantification of total DNA content after decellularization confirmed that all human LN sections had less than 48 ng DNA per section, remaining below the recommended threshold of 50 ng/mg of DNA in decellularized tissue. This threshold was met across 11 LN samples from three distinct cadaveric donors, with each condition tested using an average of three technical replicates (Fig. 6A). These findings demonstrate that our protocol achieved sufficient DNA removal in human tissue. Human FRCs seeded onto human dLN sections, following the same protocols used for murine studies, showed stable bioluminescence levels over 21 days, confirming successful human FRC engraftment onto dLN sections (Fig. 6B). Interestingly, human FRCs exhibited higher bioluminescence levels than mouse FRCs, which we hypothesized may be due to their larger cell size (Figure S4 A). To test this, we quantified cell length (Figure S4 B) and compared bioluminescence at equivalent cell numbers (Figure S4 C). Human FRCs were significantly larger and displayed higher bioluminescence levels even at matched cell numbers compared to mouse FRCs.

Human FRC-seeded dLN sections were stained for collagen I and fibroblastic markers, including α-SMA and F-actin (Fig. 6C–D) after 14 days in culture, similar to mouse studies. Immunofluorescence staining and confocal imaging confirmed the integration of seeded human FRCs within the dLN matrix. Specifically, collagen I and ECM architecture remained intact following decellularization (Fig. 6C), and F-actin⁺ cells were observed within the dLN section matrix after 14 days of culture (Fig. 6D). Collectively, these findings suggest that our protocols enable human LN sectioning and decellularization and that human dLN sections support human FRC attachment and culture.

## Discussion

The ECM plays a critical role in regulating cell behavior by providing mechanical support and biochemical cues that influence cell adhesion, migration, and differentiation^2–5^. Disruptions in ECM structure and composition are associated with cancer and autoimmune disorders^16–18,20,52,57–59^. Despite the ECM’s role in modulating cell behavior and disease, there are a lack of in vitro models that recapitulate the LN microenvironment. Current models using decellularized tissue often lack spatial resolution, rely on thick or inconsistently processed scaffolds, and are not compatible with high-resolution imaging or spatially confined recellularization^27,30,31^. To address these limitations, we developed a platform that integrates vibratome sectioning with ECM-preserving decellularization to generate thin, uniform LN scaffolds. Decellularized scaffolds have been previously used to model fibrosis^23^, asthma^24^, and kidney disease^26^. Others have employed LN scaffolds to study cancer immunotherapy^60,61^ and infection responses^62,63^, and lymph node-on-a-chip systems^64^. However, to our knowledge, we report the first protocol that combines LN sectioning and decellularization, and enables FRC reseeding and downstream analyses.

Our dual-detergent approach with SDS and Triton X-100 reduced LN DNA levels to below 50 ng/mg, the widely accepted threshold for effective decellularization^21,49^. This benchmark was met in both mouse and human LN samples, suggesting that our protocol may be used across species. We optimized SDS concentration to balance effective decellularization while preserving ECM integrity, as previously reviewed^65^. Our final protocol used 0.2% SDS for whole LNs and 0.1% SDS for LN sections, followed by 1% Triton X-100. SDS facilitates robust removal of nuclear content^49^, while Triton X-100 enhances lipid solubilization^65^. Our results show that excessive SDS not only fails to improve DNA removal but can paradoxically increase residual DNA measurements. This phenomenon is likely caused by DNA aggregation at the LN tissue surface resulting from the harsh SDS detergent^66^, which traps residual detergent and DNA fragments that are difficult to remove by washing^67^. Our findings underscore the importance of optimizing both reagent concentrations and post-decellularization washing protocols to ensure efficient tissue decellularization.

Atomic force microscopy (AFM) analysis confirmed that the elasticity of dLN sections remained comparable to that of native LN tissue, suggesting that key biophysical cues may be preserved following decellularization. Tissue stiffness is a regulator of proliferation, apoptosis, and therapeutic resistance^68^. Maintaining native tissue elasticity is therefore needed to model cell–matrix interactions in lymphoid tissues and study how mechanical cues influence cell function. The use of a micron-scale AFM probe enabled detection of local mechanical differences, capturing microscale variations in tissue architecture. One limitation of our AFM approach is that it measured elasticity across various LN regions, which may inherently possess distinct mechanical properties^69–72^. To address this, AFM measurements in this study were performed across six to ten different vibratome-sliced dLN sections, with a minimum of ten measurements recorded throughout the section, enabling broad spatial sampling and improved representation of LN mechanical variability. Despite regional differences in native LNs, the elasticity values of LN sections fell within the reported physiological range for native LN tissue^72^. Interestingly, dLN sections exhibited lower variability in elasticity compared to native LNs. This is likely due to the removal of cellular elements that exhibit regional mechanical heterogeneity^73^ from the dLN sections. Although decellularization may reduce the microscale mechanical heterogeneity of dLN sections relative to native LN tissue, the resulting uniformity provides a more consistent LN substrate. This consistency may facilitate the interpretation of elasticity values and mechanobiological studies using dLN sections across different disease states and stromal cell populations.

Our decellularization protocol retained detectable levels of ECM components of native LN tissue via immunofluorescence (Collagen I, Collagen IV, fibronectin and laminin) and quantitative assays (total collagen and GAG concentration). While other groups have reported only partial retention of glycosaminoglycans (GAGs) after SDS-based decellularization^66^, our results indicate a relative preservation of GAG content. Immunofluorescence staining and collagen quantification assays further confirmed the presence of collagen and other ECM proteins following decellularization. However, because the collagen quantification assay involves hydrolysis of collagen and thus cannot distinguish between intact and denatured forms, and because immunostaining can detect both intact and denatured collagen, additional validation would be necessary to confirm collagen structural integrity. Future studies using more specific tools, such as collagen hybridizing peptides^74^, will be essential to assess whether the collagen maintained its native conformation following decellularization.

We then sought to confirm that our dLN sections could support murine and human stromal cell culture, retention, and extraction. FRCs were chosen for seeding onto dLN sections because they build the paracortex LN ECM^41^ and respond to matrix cues^42^. FRCs seeded onto dLN sections remained viable over time, could be longitudinally monitored via bioluminescence, and were successfully extracted for downstream analysis using our digestion protocol. Murine studies confirmed that FRCs in dLN sections are viable for 28 days, while human FRCs in human dLN sections were assessed for 21 days. Previous studies have shown that, while FRC phenotypic marker expression is apparent by day 14, culture for 21 days may be necessary for scaffold remodeling to reach baseline levels established by controls^19^. Hence, we determined 21 days as both a biologically relevant and practical endpoint to assess survival in our system. Nevertheless, it is important to note that our human-derived cultures were not evaluated beyond 21 days. Future studies will be required to determine how FRC phenotype and viability change over extended periods, as other organoid systems have demonstrated stability for 60 days or longer^75–77^.

Additionally, FRCs showed expression at the protein level of fibroblastic markers (α-SMA, vimentin, F-actin) within seeded dLN sections, further suggesting their survival and integration. Because our dLN sections are compatible with high-resolution microscopy, they could enable future longitudinal analyses of cellular behavior, ECM remodeling, and marker expression within the same tissue section. While we used flow cytometry for downstream FRC characterization, our system also supports additional applications such as RNA extraction and multiplexed imaging. Given this versatility, our dLN platform could support other groups in studying how ECM cues influence immune cell behavior, differentiation, and transcriptional states.

Importantly, we observed that expression of FRC phenotypic markers gp38 and PDGFRα varied depending on both the culture conditions and mouse strain, corroborating previous work^42^. NOD-derived FRCs, which initially exhibited low gp38 expression, showed a modest increase when cultured within NOD dLN sections, whereas B6-derived FRCs maintained high gp38 levels overall but displayed reduced PDGFRα expression after 14 days. These findings highlight the phenotypic plasticity of FRCs, as reported in human FRCs^53^, and suggest that the dLN microenvironment can influence fibroblast marker expression. PDGFRα, a fibroblast marker whose expression is known to fluctuate with culture duration^56^, substrate stiffness^54,56^, and biological context^54,55^, may be dynamically regulated by local cues. Future studies could use our platform to interrogate how disease-specific LN microenvironments affect stromal cell phenotype and function. For example, dLN sections derived from different disease models (cancer or chronic inflammation) could enable mechanistic comparisons of stromal cell responses within distinct ECM contexts.

Additionally, our results demonstrate that the dLN section platform supports functional co-culture of FRCs and T cells, enabling antigen presentation by FRCs and subsequent T cell activation and proliferation. This suggests that our dLN sections could provide a physiologically relevant system to study stromal-immune cell interactions in a tissue-like context, bridging the gap between traditional 2D cultures and in vivo models. Future work could assess how FRCs regulate T cell activation depending on the dLN substrate origin. These studies will enable more complex modeling of immune cell dynamics and cell-ECM crosstalk. Importantly, the compatibility of our protocols with human LNs could support translational applications across diverse donor types and clinical states to model therapeutic responses and disease progression.

## Conclusion

We used vibratome sectioning and decellularization protocols in mouse and human LNs to generate dLN sections with preserved ECM elasticity that enable FRC culture and co-culture with T cells. Our decellularization protocol retained detectable levels of key ECM components of native LN tissue. Immunofluorescence staining and quantitative assays indicated that collagen and glycosaminoglycan (GAG) levels were comparable to those in native lymph nodes. The resulting dLN sections enabled uniform FRC seeding, supported 28-day murine FRC culture, 21-day human FRC culture, co-culture with T cells, and allowed high-resolution imaging. NOD and B6-derived FRCs were successfully extracted from dLNs for downstream flow cytometry analysis, and there were changes in the expression of the phenotypic marker gp38 and the fibroblast marker PDGFRα in FRCs cultured in dLN sections compared to standard 2D culture. Our dLN platform could be used as a versatile and physiologically relevant tool in basic and translational immunoengineering research.

## Supplementary Information

Below is the link to the electronic supplementary material.

Supplementary Material 1

## Data Availability

The datasets used and/or analyzed during the current study will be made available from the corresponding authors (L.N.T. or A.A.T.) upon reasonable request.
