# Supplementary material for: Decellularized lymph node sections with preserved extracellular matrix for stromal cell culture
Source: Sci Rep. 2025 Nov 24;16:107. doi: 10.1038/s41598-025-28782-0 (PMC12764942; doi:10.1038/s41598-025-28782-0)
Supplement: Supplementary file 1 — Supplementary Material 1 [file 41598_2025_28782_MOESM1_ESM.docx]

**Supporting Information**

**Supplementary Table 1:** Lymph Node Vibratome Sectioning Parameters

| **Parameter** | **Trial 1** | **Trial 2** | **Trial 3** | **Trial 4** | **Trial 5** | **Trial 6** | **Trial 7** | **Trial 8** |
| --- | --- | --- | --- | --- | --- | --- | --- | --- |
| **Agarose** | 3% | 6% | 6% | 6% | 8% | 8% | 8% | 6% |
| **Velocity** | 1 mm/s | 1 mm/s | 1 mm/s | 1.5 mm/s | 1 mm/s | 1.5 mm/s | 1 mm/s | 1 mm/s |
| **Thickness** | 120 μm | 120 μm | 150 μm | 150 μm | 150 μm | 200 μm | 200 μm | 200 μm |
| **Recovery Rate** | 20% | 30% | 40% | 40% | 50% | 70% | 75% | 75% |

**Supplementary Table 2:** FRC Extraction Protocols and Resulting Viability

| **Extraction Method** | **1** | **2** | **3** | **4** | **5** |
| --- | --- | --- | --- | --- | --- |
| **Reagent** | Enzyme Mix 1 mL | Trypsin  1 mL | Enzyme Mix 3 mL | Trypsin  3 mL | Enzyme Mix  1 mL |
| **Mechanical Disruption of Sections** | No | No | No | No | Yes |
| **Incubation time** | 3 min | 5 min | 5 min (repeat) | 5 min | 3 min |
| **Vortex time** | 30 s | 0 min | 30 s (repeat) | 30 s | 5 s on low setting |
| **Total Cell Count** | 33,100 cells | 5,510 cells | 121,000 cells | 6,100 cells | 210,000 cells |
| **Live Cell Count** | 11,000 cells | 5,510 cells | 16,500 cells | 0 | 138,000 cells |
| **Viability** | 33% | 100% | 14% | 0% | 66% |

**
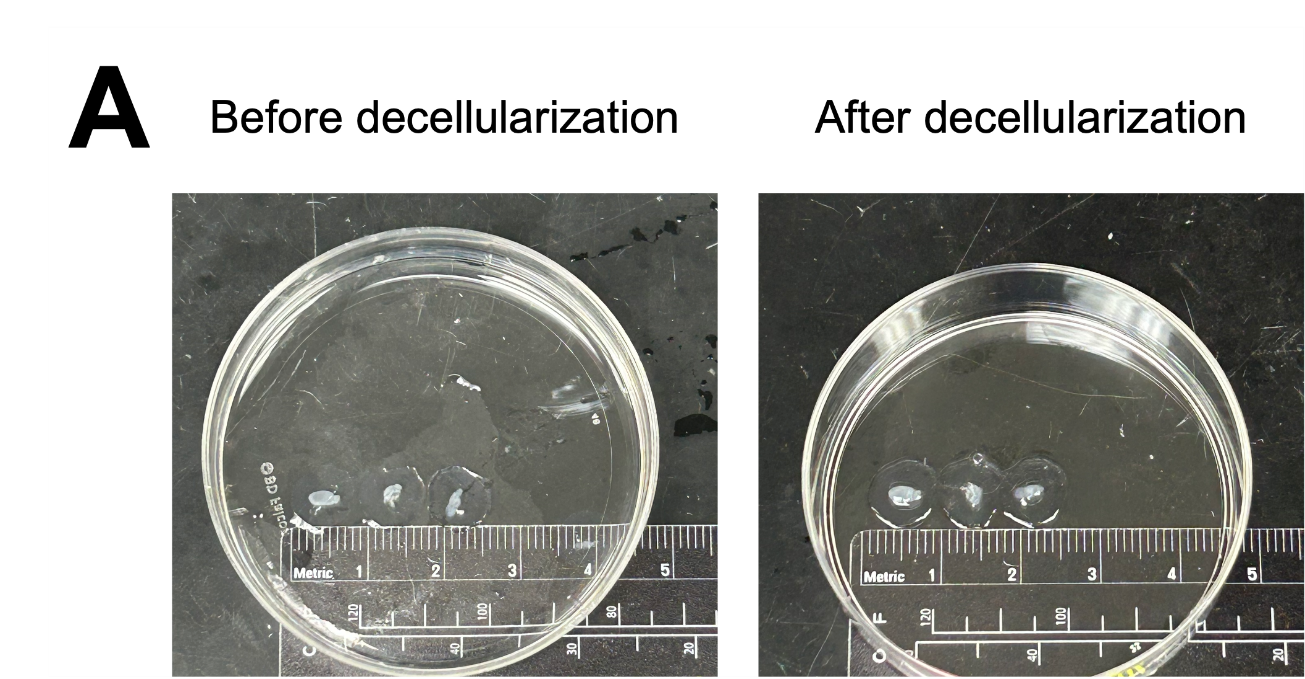
**

**Figure S1:** Representative images of mouse lymph node sections before and after decellularization.

**
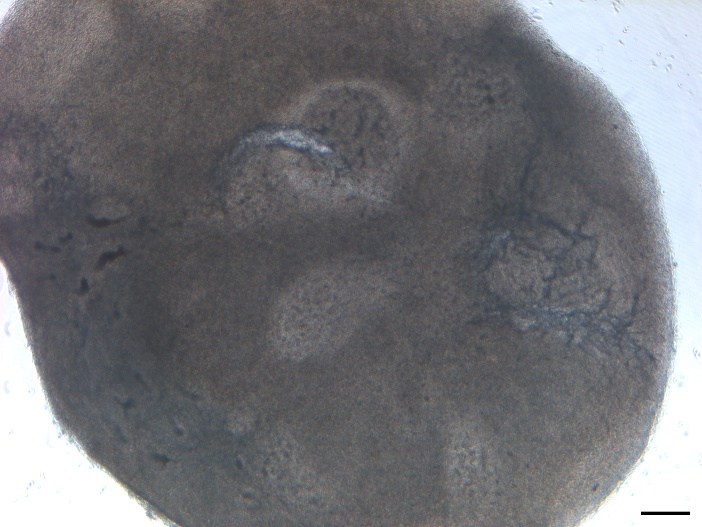
**

**Figure S2**: Representative light microscopy image of a decellularized lymph node section seeded with FRCs and cultured for 14 days. Scale bar = 200 μm.

**
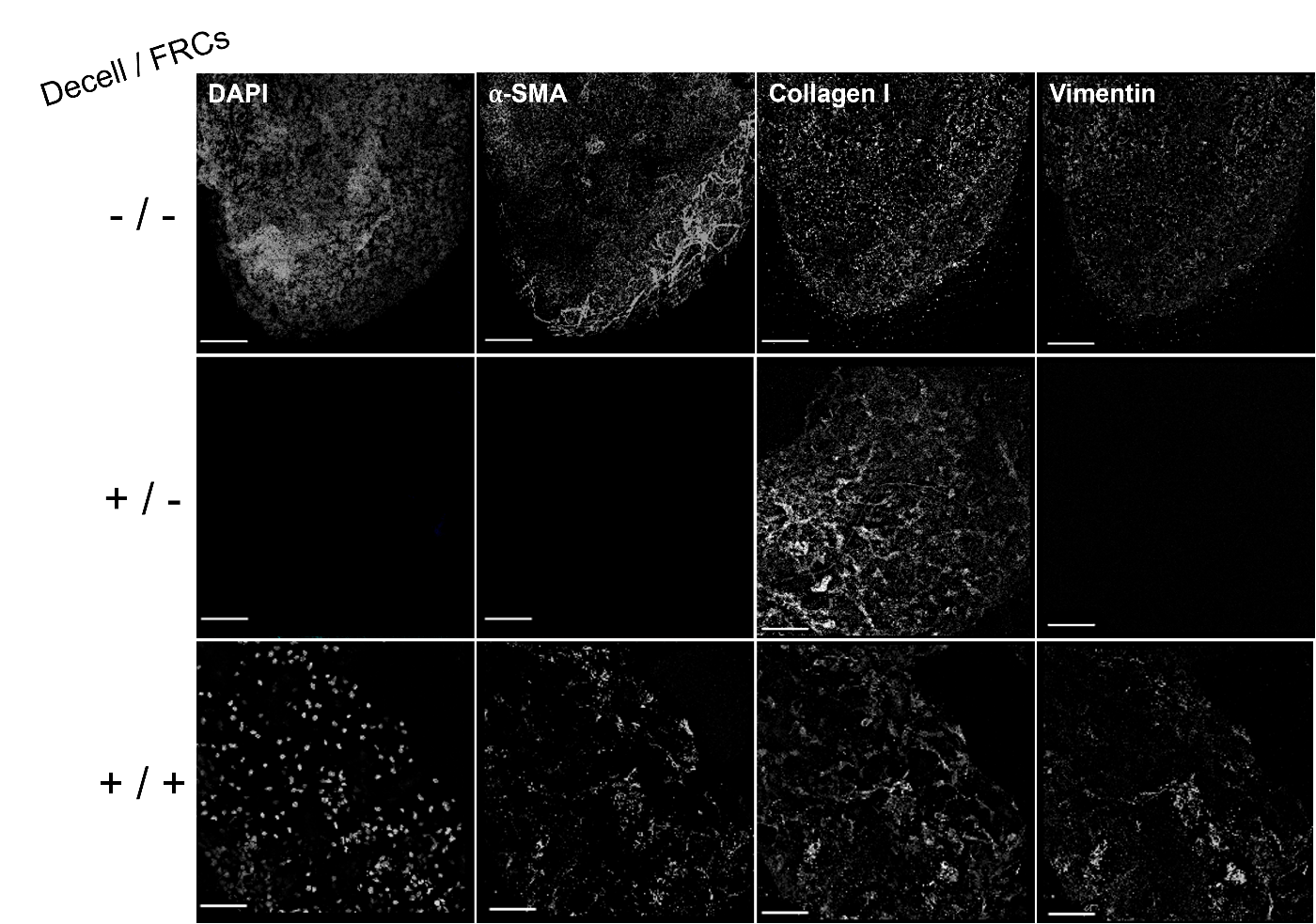
**

**Figure S3:** Representative immunofluorescence images of control non-decellularized LN section (-/-), dLN sections before cell seeding (+/-), and dLN sections seeded with FRCs (+/+) and cultured for 14 days, stained for DAPI, fibroblastic marker α-SMA, ECM marker collagen I, and cytoskeletal marker vimentin. Scale bar = 100 μm. Decell = Decellularized; FRCs = Seeded with FRCs.


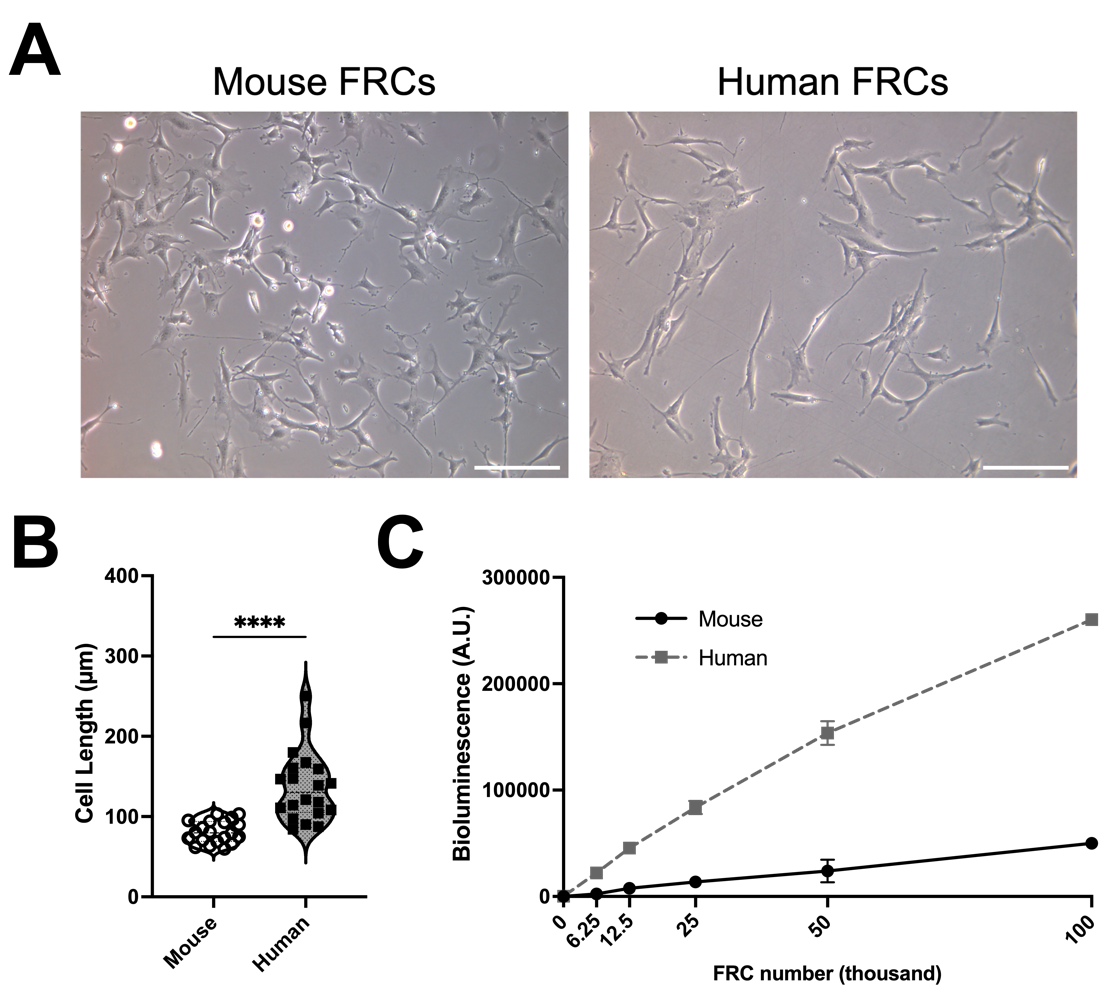


**Figure S4: (A)** Representative light microscope images of mouse (left) and human (right) FRCs. Scale bar = 200 μm. **(B)** Quantification of cell length, measured as the longest distance between two points along the fibroblast fibers using ImageJ ‘Measure’ tool. **(C)** Bioluminescence levels of mouse (black) and human (grey) FRC as a function of cell numbers via RealTime-Glo™ assay. Data represented as mean ± standard deviation. P-values were determined by unpaired, student’s t-test (****p < 0.0001).
